# Can ^18^F-FDG PET/CT Radiomics Features Predict Clinical Outcomes in Patients with Locally Advanced Esophageal Squamous Cell Carcinoma?

**DOI:** 10.3390/cancers14123035

**Published:** 2022-06-20

**Authors:** Vetri Sudar Jayaprakasam, Peter Gibbs, Natalie Gangai, Raazi Bajwa, Ramon E. Sosa, Randy Yeh, Megan Greally, Geoffrey Y. Ku, Marc J. Gollub, Viktoriya Paroder

**Affiliations:** 1 Molecular Imaging and Therapy Service, Department of Radiology, Memorial Sloan Kettering Cancer Center, New York, NY 10065, USA; jayaprav@mskcc.org (V.S.J.); yehr@mskcc.org (R.Y.); 2 Department of Radiology, Memorial Sloan Kettering Cancer Center, New York, NY 10065, USA; pgibbs1966@gmail.com (P.G.); gangain@mskcc.org (N.G.); bajwar@mskcc.org (R.B.); sosar@mskcc.org (R.E.S.); gollubm@mskcc.org (M.J.G.); 3 Mater Private Hospital, D07 WKW8 Dublin, Ireland; megan.greally@materprivate.ie; 4 Department of Medicine, Memorial Sloan Kettering Cancer Center, New York, NY 10065, USA; kug@mskcc.org

**Keywords:** esophageal squamous cell carcinoma, PET/CT, radiomics, progression-free survival, overall survival

## Abstract

**Simple Summary:**

PET/CT is an important staging modality in the baseline assessment of locally advanced esophageal squamous cell carcinoma. Accurate staging and response prediction in these patients is essential for management. The aim of this retrospective study was to assess the usefulness of ^18^F-FDG PET/CT radiomics features in predicting outcomes such as tumor and nodal categories, PET-based response to induction chemotherapy, progression-free survival, and overall survival. In a final cohort of 74 patients, we found that the developed radiomics models can predict these clinical and prognostic outcomes with reasonable accuracy, similar or better than those derived from conventional imaging. Future studies with a larger cohort would be helpful in establishing the significance of these models.

**Abstract:**

This study aimed to assess the usefulness of radiomics features of ^18^F-FDG PET/CT in patients with locally advanced esophageal cancers (ESCC) in predicting outcomes such as clinical tumor (cT) and nodal (cN) categories, PET response to induction chemotherapy (PET response), progression-free survival (PFS), and overall survival (OS). Pretreatment PET/CT images from patients who underwent concurrent chemoradiotherapy from July 2002 to February 2017 were segmented, and data were split into training and test sets. Model development was performed on the training datasets and a maximum of five features were selected. Final diagnostic accuracies were determined using the test dataset. A total of 86 PET/CTs (58 men and 28 women, mean age 65 years) were segmented. Due to small lesion size, 12 patients were excluded. The diagnostic accuracies as derived from the CT, PET, and combined PET/CT test datasets were as follows: cT category—70.4%, 70.4%, and 81.5%, respectively; cN category—69.0%, 86.2%, and 86.2%, respectively; PET response—60.0%, 66.7%, and 70.0%, respectively; PFS—60.7%, 75.0%, and 75.0%, respectively; and OS—51.7%, 55.2%, and 62.1%, respectively. A radiomics assessment of locally advanced ESCC has the potential to predict various clinical outcomes. External validation of these models would be further helpful.

## 1. Introduction

Positron emission tomography/computed tomography (PET/CT) with 18-fluorine-labeled fluorodeoxyglucose (^18^F-FDG) has become an established modality of investigation in the staging of patients with esophageal cancer, providing incremental information leading to changes in management for up to one third of patients [1]. It has been shown to provide valuable prognostic information prior to any treatment and during and after chemoradiotherapy [2,3]. Correlations between prognosis and various metabolic parameters such as standardized uptake value, total lesion glycolysis, and metabolic tumor volume have been reported in several studies [4,5,6,7]. Radiomics analysis allows us to use the diagnostic images further by extracting information otherwise “invisible” to the naked eye and thus potentially improve the diagnostic and prognostic accuracy of a given study [8,9]. It follows a complex workflow which includes image acquisition, preprocessing, region of interest (ROI) segmentation, feature extraction, and feature analysis [10]. In a meta-analysis, Park et al. highlighted that almost 90% of the radiomics analyses were conducted for oncological studies, and mainly utilized for diagnosis and grading of the tumor, molecular biology and genomics assessment, predicting survival outcomes, and treatment response [11].

In an overview of the currently available imaging biomarkers in upper gastrointestinal cancers, Gabelloni et al. concluded that various imaging biomarkers and radiomic features provide significant additional information to conventional imaging parameters that can guide the management of these patients at all stages [12]. In recent years, there has been increasing interest in the radiomics analysis of esophageal cancers for prediction of extent of disease and response to treatment. Most of these studies predominantly focus on predicting response to treatment [13,14,15]. By contrast, very few studies have looked at clinical outcome parameters such as nodal status, tumor stage, overall survival (OS), or progression-free survival (PFS) [16,17,18,19,20]. Low sample size is also one of the limiting factors in assessment of radiomics signatures in many esophageal cancer studies. It should also be noted that there are significant differences in tumor biology, characteristics, and prognostic features between esophageal squamous cell carcinoma (ESCC) and adenocarcinoma, and as such these should be treated as different diseases.

We hypothesized that, in a specific subset of esophageal cancer patients, i.e., those with locally advanced ESCC without distant metastases, the radiomics features of ^18^F-FDG PET/CT could provide valuable information regarding various clinical outcome parameters which might inform further management. Thus, the purpose of this study was to investigate various radiomics features on CT, PET, and combined PET/CT image datasets to predict clinical tumor and nodal categories as defined by the American Joint Committee on Cancer (AJCC), PFS, and 3-year OS in patients with locally advanced ESCC without distant metastases.

## 2. Materials and Methods

### 2.1. Patient Inclusion

**Figure 1:**
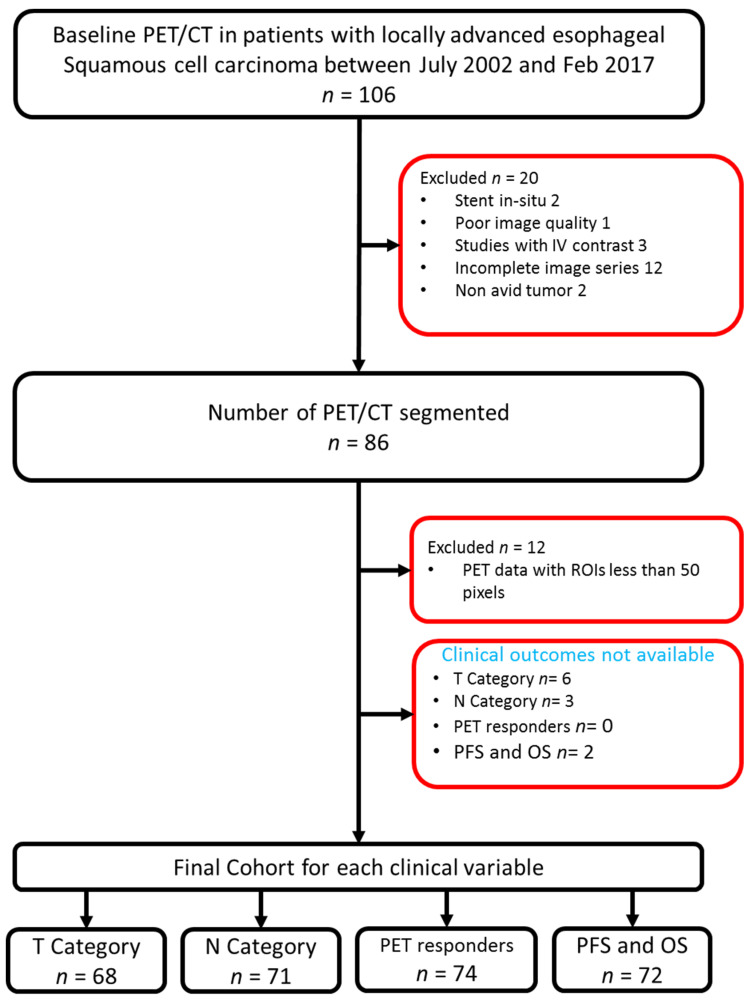
Flowchart of patient inclusion in the study. Abbreviations: T, clinical tumor category; N, clinical nodal category; PFS, progression-free survival; OS, overall survival.

This was a retrospective, Health Insurance Portability and Accountability Act-compliant study with approval from our institutional review board and a waiver for written informed consent. Patients with locally advanced ESCC, without distant metastases, and who underwent induction chemotherapy followed by concurrent chemoradiation at our institution between July 2002–February 2017 were included in the study. The sample was derived from a prior study which evaluated post-induction chemotherapy PET/CT for predicting outcomes in the patients with ESCC who received chemoradiation [2]. Of the 106 patients initially identified, 20 patients were excluded due to esophageal stents in situ, PET/CTs performed with intravenous contrast, poor image quality due to increased background statistical noise, and non-FDG-avid primary tumors. A total of 86 patients were included for tumor segmentation. A flowchart of patient inclusion to the study is presented in Figure 1.

### 2.2. Treatment and Imaging

All patients underwent a pretreatment baseline PET/CT and another PET/CT following induction chemotherapy. Patients received a variety of platinum-based induction chemotherapy regimens and the post-induction PET/CT was performed within a median of 8 days (range, 1–32 days) from completion of induction chemotherapy. Patients with a minimum of 35% decrease in maximum standard uptake value (SUVmax) in the primary tumor after induction chemotherapy were considered as responders [3,21]. Those patients who were considered responders continued with the same chemotherapy during radiation. Of the 25 patients who were considered PET non-responders, 16 patients were continued on the same chemotherapy during chemoradiation, and the remainder were changed to alternate chemotherapy. The decision to perform surgery was based on individual cases. In this analysis, the PET non-responders were analyzed as a single group, irrespective of whether they continued with or changed chemotherapy during radiation.

### 2.3. Image Acquisition and Segmentation

The study included baseline PET/CTs that were performed either at our institution or an outside institution. Scanners and PET/CT acquisition parameters are presented in Table 1. Segmentations were performed on PET and CT separately, using Hermes Gold LX software version 2.9.1.0, Stockholm, Sweden, by two radiologists with 8 and 10 years of experience in oncological imaging, blinded to each other’s contours and to clinical information (VP and VSJ). The PET/CT images were analyzed on the Hermes Hybrid 3D software version 3.0.1, Stockholm, Sweden. The tumor was first identified by the readers on the attenuation-corrected PET images and a constraint ROI was manually drawn around the tumor. An automatic target volume was then generated within the constraint ROI using a threshold tool in Hermes Hybrid 3D software set to “Hot mode” with a minimum default of 2.5 SUVbw. This threshold tool uses an isocontour around a collection of a volume of voxels generated by a mathematical rule. In the Hermes software, “Hot mode” selects the values inside the isocontour that are greater than a threshold value. In this case, we used a value of SUVbw 2.5. Ideally, this means that the minimum set is the value of all the pixels on the edge, but due to discrete values, it should be close. Once the automatic volume was generated by the Hermes software, individual readers corrected the delineated segments to remove what was subjectively judged (based on their own clinical experience) to be inflammatory uptake along the proximal and distal edges of the tumor. On CT, the esophagus was manually segmented at the levels of the tumor identified on PET and the voxels representing air were excluded from analysis. Paraesophageal lymph nodes distinct from the primary tumor with a clear fat plane were excluded from the segmented volume. Twenty cases were segmented by both radiologists to assess inter-reader agreement.

### 2.4. Radiomics Analysis

Segmented volumes were exported to MATLAB (version 9.3.0.713579 (R2017B), The MathWorks, Inc., Natick, MA, USA) for feature extraction. All images were interpolated to the median in-plane spatial resolution (0.977 mm for CT data and 5.31 mm for PET data) prior to radiomics analysis. The CT images were reduced to 64 gray levels prior to radiomics feature calculations. Because of the small pixel count due to reduced spatial resolution, the PET data were reduced to 16 gray levels only; this also accounted for institutional differences in PET acquisition. To ensure adequate counting statistics for radiomics feature calculations, PET data with regions of interest (ROIs) of 50 or more pixels only were included. Radiomics features were calculated using CERR, which has been shown to be compatible with the emerging image biomarker standardization initiative [22]. One hundred and one features were calculated in six classes (22 first-order (FO), 26 based on gray-level cooccurrence matrices (GLCM), 16 based on run-length matrices (RLM), 16 based on size-zone matrices (SZM), 16 based on neighborhood gray-level dependence matrices (NGLDM), and 5 based on neighborhood gray-tone difference matrices (NGTDM)). The data were initially split into separate training and test sets (60:40 split). All model development was performed on the training set and the test set was reserved solely for determination of final diagnostic metrics. Class imbalances in the training data were removed by employing adaptive synthetic sampling to equalize class sizes [23]. This was performed to prevent subsequent models potentially classifying all cases as belonging to the majority class. An elastic net, combining ridge and LASSO regression, was then utilized to determine which coefficients (radiomics features) were of most importance. A maximum of 5 features were selected to avoid overfitting. If fewer features were determined to be of importance, only those were forwarded for use in model development. Models were considered utilizing CT radiomics data alone, PET radiomics data alone, and then CT and PET radiomics data combined. Predictive models were then developed in MATLAB using support vector machines and 5-fold cross-validation. The developed models were than investigated using the test dataset to determine the final diagnostic accuracies. A similar method employing separate training and test datasets, with predictive models developed using cross-validation (in this case nested cross-validation) on the training data, has been demonstrated in breast cancer [24].

Clinical parameters such as the clinical tumor and nodal categories and pathologic complete response were recorded via the electronic medical records. PET responders were calculated based on more than a 35% decrease in SUVmax values of the primary tumor on post-induction PET/CT compared to the baseline study [6]. OS was calculated from the date of post-induction PET/CT to the date of death. Patients who were alive and did not experience an event were censored at the date of last follow-up. Date of progression was based on either histology or imaging features consistent with recurrence or metastatic disease. PFS was calculated from the date of the post-induction PET/CT to the date of progression or death, whichever occurred first. The cases were classified into binary categories as follows: T2 vs. T3/4, N0 vs. N1/2, PET responders vs. non-responders, PFS “yes” or “no”, and 3-year OS “yes” or “no”. Response outcomes were predicted using these binary classifiers.

### 2.5. Statistical Analysis

For all the final predictive models, diagnostic metrics including sensitivity, specificity, positive predictive value, negative predictive value, accuracy, and area under the curve (AUC) were calculated using MedCalc for Windows, version 15.0 (MedCalc software, Ostend, Belgium) and compared with the test dataset using McNemar’s test.

Inter-reader agreement was determined using Jaccard indices (intersection size over union size) and dice similarity coefficients (twice the intersection size over the sum of the two individual regions) using MATLAB. A two-way mixed-effects model with a single measure was used to judge the intraclass correlation coefficient. Results from these two metrics were interpreted as follows: values < 0.40 = poor agreement, 0.41–0.59 = fair agreement, 0.60–0.79 = good agreement, and 0.80–1.00 = excellent agreement. Only parameters with good or excellent agreement were considered for subsequent predictive model development. As a result of ICC analysis, 11 CT radiomics features and 3 PET radiomics features were excluded from further analysis (Supplementary Materials Table S2).

## 3. Results

### 3.1. Patient Characteristics

**Figure 2:**
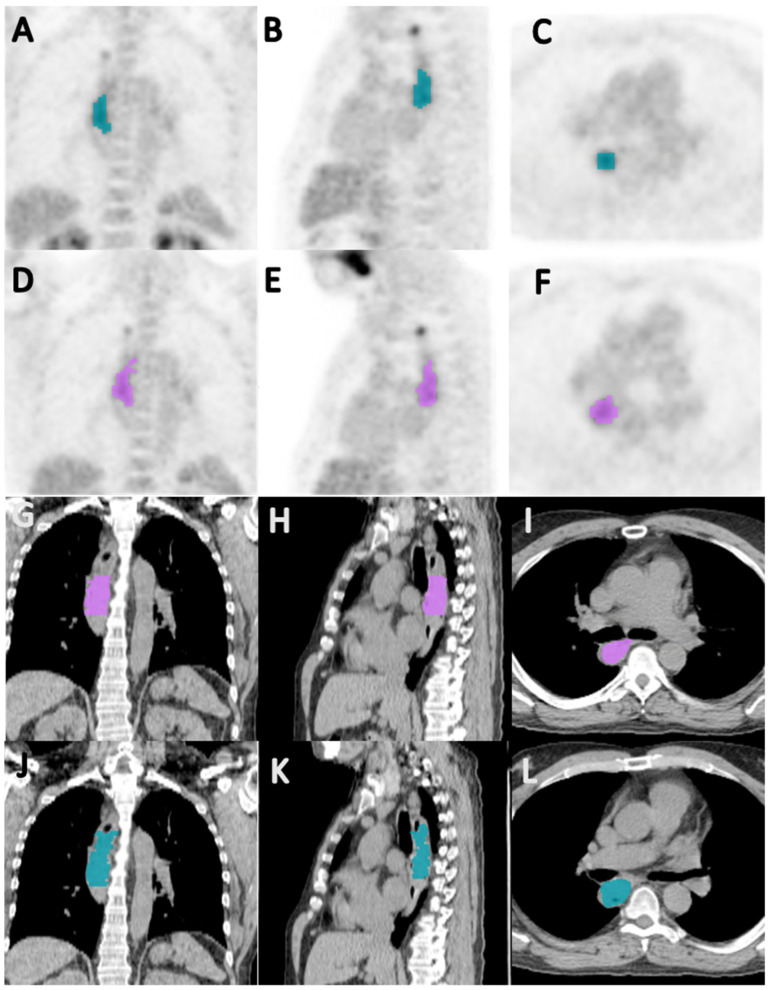
Coronal (**A**,**D**), sagittal (**B**,**E**), and axial (**C**,**F**) PET images, and coronal (**G**,**J**), sagittal (**H**,**K**), and axial (**I**,**F**) CT images showing segmentation of primary tumor by reader 1 (**A**–**C**,**G**–**I**) and reader 2 (**D**–**F**,**J**–**L**), in a 64-year-old male patient with esophageal squamous cell carcinoma; the Jaccard index and a dice similarity coefficient for inter-reader agreement on the segmented volumes were 0.657 and 0.793, respectively.

Baseline PET/CTs were segmented in 86 patients (58 men, 28 women) with a mean age of 65 years (range, 41–87). A total of 12 patients were excluded from the analysis due to small lesion size (<50 pixels), leaving a maximum of 74 patients for analysis. The clinical outcomes were dichotomized and the patients whose clinical outcomes were not known were excluded from respective assessments. Although there were several chemotherapy combinations, virtually all were platinum-based and nearly 92% of the patients received either platinum/paclitaxel- or platinum/irinotecan-based chemotherapy. Patient characteristics are presented in Table 2. There was good to excellent inter-reader agreement with the average Jaccard indices for the CT and PET data being 0.64 and 0.75, respectively, and the average dice similarity coefficients for the CT and PET data being 0.77 and 0.85, respectively (Figure 2).

### 3.2. Diagnostic Accuracy of CT, PET, and Combined PET/CT Training and Test Datasets for Various Clinical Parameters

The total number of patients included in each category, as well as classification into training and test datasets, is presented in Table 3. Diagnostic accuracy of CT, PET, and combined PET/CT training and test datasets for various clinical parameters is presented in Table 4. Radiomics features used for the model development and all diagnostic metrics obtained for the training and test dataset are presented in the Supplementary Materials Tables S3–S8.

**Figure 3:**
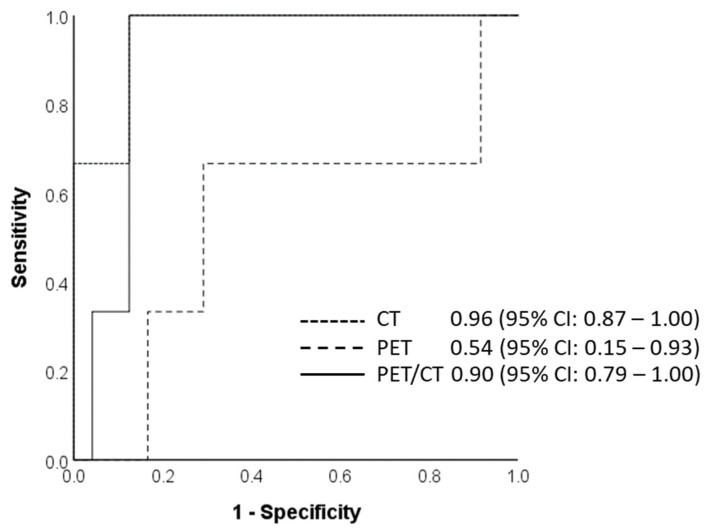
Area under the curve for the final radiomics model to predict tumor category on CT, PET, and PET/CT test datasets using five radiomics features, calculated at 0.96, 0.54, and 0.90, respectively; 95% confidence intervals are presented within parentheses.

For the prediction of tumor category, the AUC of the training datasets for CT, PET, and combined PET/CT was 0.89, 0.90, and 0.87, respectively (Figure 3). The diagnostic accuracies of the test dataset for the prediction of tumor category were similar for CT, PET, and combined PET/CT datasets (70.4%, 70.4%, and 81.5%, respectively; *p*-value CT vs. PET/CT = 0.219, PET vs. PET/CT = 0.289, CT vs. PET = 1.000). The AUC for the CT and combined PET/CT test datasets was above 90% (0.96 for CT test and 0.90 for the PET/CT test). All three models, however, demonstrated a poor negative predictive value.

**Figure 4:**
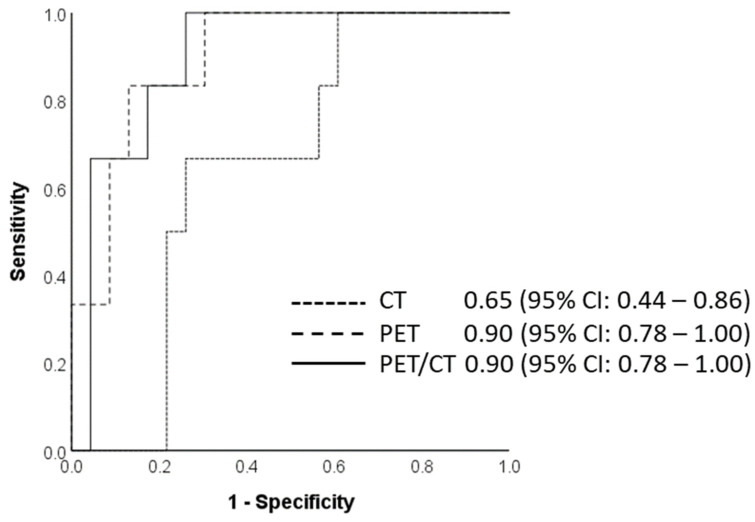
Area under the curve for the final radiomics model to predict N category on CT, PET, and PET/CT test datasets using five radiomics features, calculated at 0.65, 0.90, and 0.90, respectively; 95% confidence intervals are presented within parentheses.

For the prediction of nodal status, the AUC for the CT, PET, and PET/CT training datasets was 0.75, 0.98, and 0.93, respectively. The PET and the combined PET/CT test datasets showed higher accuracy (86.2% each) and had a similar AUC of 0.90 compared to the CT test dataset (Figure 4). There was no improvement in the accuracy when CT or PET datasets were compared to the PET/CT datasets (*p*-value CT vs. PET/CT = 0.109, PET vs. PET/CT = 1.000). There was, however, a trend towards significance between the PET and the CT data (*p*-value = 0.070). The combined PET/CT dataset had a better specificity than the PET dataset alone (66.7% vs. 33.3%).

Response assessment based on percentage reduction of SUVmax on the post-induction PET/CT has shown to predict disease-free survival and overall survival [6]. The training dataset for the prediction of PET response demonstrated an AUC of 0.68, 0.77, and 0.84 for the CT, PET, and combined PET/CT radiomics models, respectively. For the test datasets, the radiomics model predicted a 70.0% diagnostic accuracy of the combined PET/CT data, which was better than the CT or PET alone (60.0% and 66.7%, respectively). The sensitivity and the positive predictive value for the combined PET/CT data were 75.0% and 79.0%, whereas the negative predictive value was only 54.6%.

**Figure 5:**
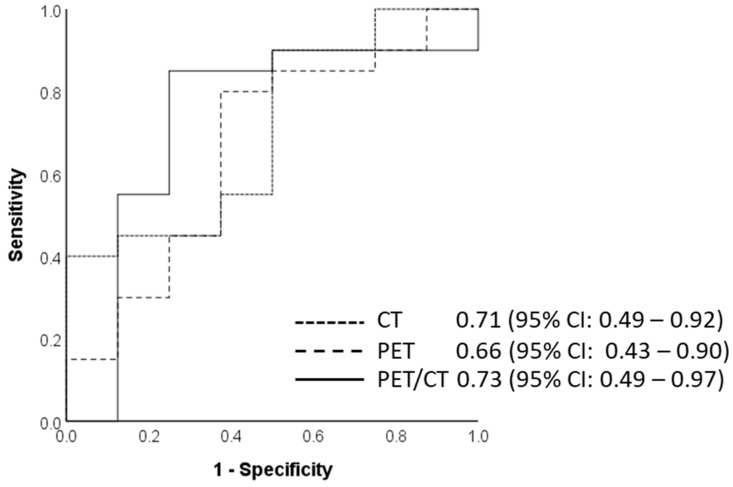
Area under the curve for the final radiomics model to predict progression-free survival on CT, PET, and PET/CT test datasets using five radiomics features, calculated at 0.71, 0.66, and 0.73, respectively; 95% confidence intervals are presented within parentheses.

The radiomics models for the CT, PET, and combined PET/CT training sets predicting PFS had an AUC of 0.65, 0.81, and 0.81, respectively (Figure 5). The PET and the combined PET/CT test data in this category showed similar specificity, negative predictive value, and diagnostic accuracy of 85.0%, 81.0%, and 75.0%, respectively. The radiomics model predicting OS had a diagnostic accuracy for both training and test datasets ranging from 51.7 to 68.0% (Table 4).

## 4. Discussion

In this study, we extracted radiomics features from CT, PET, and combined PET/CT datasets in patients with locally advanced ESCC to predict various clinical outcomes such as tumor and nodal categories, PET responders to induction chemotherapy, PFS, and OS. The diagnostic accuracies of the CT test datasets for tumor and nodal categories, PET response, and PFS were between 60.0 and 70.4%. The PET and the combined PET/CT dataset radiomics models for these clinical outcomes demonstrated diagnostic accuracies from 66.7 to 85.7%, and from 75.0 to 86.2%, respectively. The diagnostic accuracy of all three models for predicting OS was between 51.7 and 68.0%.

Our radiomics model based on CT, PET, and PET/CT for predicting the clinical tumor (cT) category performed well with a diagnostic accuracy of over 70%. Although the AUC for the PET data was low in our study, the combined PET/CT dataset had an AUC of 0.90 with an accuracy of 81.5% (95% CI, 61.9–93.7%). Despite the low negative predictive value, which could be a reflection of low numbers in the minority class, our results demonstrate that the developed radiomics model has the potential to differentiate early and late cT-category ESCC. The final model incorporating both CT and PET radiomics features utilized four features from CT and only one from PET, suggesting that CT data are more important in this circumstance. Two of the first three selected features were derived from size-zone matrices, indicating that the assessment of zones with similar intensities is key here. Busyness measures changes in gray levels between neighboring voxels; thus, the ROI looking “busy” was the second most important feature selected.

Regarding the prediction of the cT category in patients with esophageal cancers, currently, endoscopic ultrasound is considered the most useful tool, although the accuracy depends on the stage. In a meta-analysis of 44 studies, the overall diagnostic accuracy of endoscopic ultrasound was reported to be 0.79 (95% CI: 77–80) with a relatively better performance in T1 substaging and T4 disease, whereas the CT-based diagnostic accuracy for the T category was 0.59 (95% CI: 54–64) [25]. Our CT test model showed an AUC of 0.96 (95% CI 0.87–1.00). The results were slightly better than the radiomics model developed by Yang et al. (AUC 0.857; 95% CI 0.691–1.000) [26]. In another study, Wu et al. used a radiomics approach to identify early- and late-stage ESCC prior to surgery [17]. The group demonstrated a significant discrimination between stages I–II and stages III–IV with an AUC of 0.795 (95% CI: 0.714−0.875) in the primary cohort and 0.762 (95% CI: 0.600−0.924) in the validation cohort. To our knowledge, prediction of the cT category based on PET or combined PET/CT radiomics models has not been reported in the literature before. T staging on PET/CT based on visual parameters alone is known to be poor. Mantziari et al. utilized FDG PET/CT-derived metabolic parameters such as maximum standardized uptake value (SUVmax), total lesional glycolysis (TLG), and metabolic tumor volume (MTV) to predict preoperative cT staging, reporting that higher SUVmax and TLG were found to be associated with cT3/T4 categories [27]. However, in clinical practice, it is very difficult to ascribe a particular cut-off value of metabolic parameters to ascertain the cT stage. Our results, as well as a very limited number of radiomics studies currently available in the literature, suggest that radiomics features could provide this information with reasonable accuracy.

Regarding the prediction of the cN category, the PET and the combined PET/CT test datasets in our study were revealed to be the best models for predicting this category, with a diagnostic accuracy of 86.2% (95% CI 68.3–96.1) and AUC of 0.90 (95% CI; 0.78–1.00), respectively. Radiomics features selected here include minimum intensity from the PET image, suggesting low uptake is important. This is further reinforced by the selection of the 10th percentile from the PET data. Here, four out of the five selected features were derived from PET data. Complexity, as calculated from the NGTDM which quantifies non-uniformity and rapid changes in gray levels, also appears to be of major importance in the prediction of the cN category. The CT test data showed a relatively lower diagnostic accuracy compared to PET and combined PET/CT data, with an AUC of 0.65 (95% CI 0.44–0.86). This was, however, still better than the accuracy of around 55% demonstrated by conventional analysis on CT or 57% on PET/CT [28]. A radiomics nomogram incorporating five features developed by Tan et al. significantly exceeded the AUC compared to size criteria alone: AUC 0.77 (95% CI 0.67–0.88) vs. 0.59 (95% CI 0.49–0.69) [18]. In another radiomics study, Wu et al. described a multilevel CT radiomics model with addition of computer vision (CV) and deep radiomics signature into clinical risk factors, which improved the prediction of lymph nodal metastasis in patient with ESCC [16]. Shen et al. also developed a predictive model for prediction of preoperative esophageal cancer lymph node metastases incorporating the radiomics signature, CT-reported suspicious lymph node number, and tumor position, although the study used a mixed cohort of adenocarcinoma (ADC) and ESCC patients [29]. Yet, prior to our study, radiomics features for PET and combined PET/CT had not been assessed before. In our study, the PET- and combined PET/CT-based models performed better than the CT-based radiomics models, suggesting the potential usefulness of radiomics analysis in predicting the nodal stage.

No radiomics studies in the past have developed a model to predict PET responders to induction chemotherapy. The MUNICON phase II study in gastroesophageal junction adenocarcinoma confirmed the significance of early metabolic response evaluation to chemotherapy and showed the feasibility of a PET-guided algorithm for treatment modification [3]. Meanwhile, Chhabra et al. demonstrated that the baseline and post-induction PET metrics were prognostic for overall survival in patients with ESCC [6]. In another study, Greally et al. conducted research based on the hypothesis that changing to different chemotherapy during radiation would salvage the PET non-responders; however, in their study, all PET non-responders had the same poor outcome, irrespective of whether they continued with the same chemotherapy regimen during radiation or were changed to an alternative chemotherapy [2]. Our combined PET/CT radiomics model predicted a response to induction chemotherapy with 35% or more decrease in SUVmax with an accuracy of 70% (95% CI 50.6–85.3) and performed better than the CT or PET models alone. The model combining CT and PET radiomics features utilized two parameters from CT data and three from PET data, suggesting that both imaging models are important here. The first selected feature was coarseness, which is an inverse measure of the level of the spatial rate of change in intensity, derived from CT data, and the second selected feature was the minimum value from PET data.

For predicting PFS, our radiomics models from the PET and combined PET/CT dataset showed an accuracy of 75% (95% CI 55.1–89.3). In the literature, assessment of conventional parameters on PET/CTs for predicting PFS has been shown to be difficult. PFS decreases with increased SUVmax at the initial PET/CT [30]. In a systematic review of 16 studies, the pooled hazard ratio (HR) of the MTV and TLG for event-free survival based on pretreatment PET/CTs was 2.03 (95% CI 1.66–2.49) and 2.57 (95% CI 1.82–3.62), respectively [31]. In a mixed cohort of ADC and ESCC, intra-tumoral heterogeneity was also shown to be associated with decreased PFS (HR, 10.78; 95% CI 1.31–88.96) [32]. Qiu et al. investigated a pretreatment CT radiomics nomogram incorporating eight radiomics features and clinical risk factors to predict postoperative recurrence risk in patients with ESCC who achieved complete pathological response after neoadjuvant chemoradiotherapy followed by surgery [33]. The nomogram yielded a C-index of 0.72 (95% CI 0.70–0.75) in the validation cohort, which was significantly better than those derived from a radiomics signature or the clinical nomogram alone (*p* < 0.0001 for each comparison) [33]. Luo et al. also developed and validated a model based on pretreatment CT radiomics features and clinical parameters to predict PFS [34]. Using 17 radiomics features, the nomogram in that study demonstrated a C-index of 0.72 (95% CI 0.65–0.79) in the validation cohort. Our radiomics model from the PET and PET/CT datasets showed similar results, although we used only five features for model development. The results from our CT datasets were relatively lower compared to others (AUC 60.7; 95% CI 40.6–78.5).

The prediction of 3-year OS in our datasets was lower than that of the other clinical outcomes studied. However, our results were similar to the random forest model based on CT radiomics developed by Larue et al. with an AUC of 0.61 (95% CI 0.47–0.75) [19]. Their study was based on the mixed cohort of both ADC and ESCC patients. Lu et al. also showed a similar result in predicting OS in patients with ESCC based on a CT tumor radiomics signature (C-Index 0.63, 95% CI 0.578–0.69), although the nomogram based on the radiomics and clinicopathological risk factors in their study showed a slightly better prediction with a C-index of 0.73 (95% CI 0.69–0.78) [35]. A CT subregion-based radiomics survival prediction model developed by Xie et al. had a similar C-index (0.71; 95% CI 0.63–0.78) [20].

For both PFS and OS, the first two selected features were derived from PET data, potentially suggesting that images from this modality are more informative in this situation. In the case of PFS, these two features were difference variance (GLCM-derived), which is a measure of heterogeneity that places higher weights on differing intensity level pairs and energy (NGLDM-derived), which assesses image homogeneity. For OS, these two features had gray-level variance (SZM-derived), which quantifies the variance in zone counts for different gray levels and cluster prominence and which determines the asymmetry of the GLCM from which it is calculated.

For all clinical outcomes, it is evident that data from both CT and PET images contributed to the classification accuracy, emphasizing the utility of both modalities. It is also apparent that calculating features from all six classes (first-order, GLCM, RLM, SZM, NGLDM, and NGTDM-based) is beneficial. Whilst it is difficult to demonstrate equivalence between individual features and human eye observation, the prevalence of second- and high-order features in the final models indicates that complex image heterogeneity, reflecting underlying tumor heterogeneity, is a major driver in clinical outcomes.

Conventional cross-sectional imaging in pretreatment evaluation of the ESCC suffers from low sensitivity and specificity in terms of clinical staging and outcome predictions. ^18^F-FDG PET/CT is now a standard of care for the management of these patients. Although the metabolic parameters SUVmax, TLG, and MTV can help in risk stratification of patients to a certain extent, there is an unmet need for better disease staging and prognostic assessment. Compared to other malignancies such as rectal or breast cancers, radiomics analysis of the esophageal cancer is still in its early stages. Some of these early studies combined patients with adenocarcinoma and those with ESCC, although adenocarcinoma and ESCC have been proven to be two distinct entities in terms of tumor biology, clinical characteristics, and response to treatment, as well as prognostic features [36]. Hence, the results from these studies should be interpreted with caution. One also has to consider the quality of the radiomics assessment performed and avoid overfitting or overparameterization. We limited our study to patients with squamous cell carcinomas only, as well as excluded patients with small lesions (<50 pixels), as texture analysis requires good statistical counting. Radiomics assessment of tumor and nodal categories on PET or combined PET/CT data has not been performed before. Radiomics analysis of PET responders is also uncharted territory. Our results demonstrate that radiomics-based analysis of ^18^F-FDG PET/CT can predict clinical outcomes and prognostic factors better than the conventional cross-sectional imaging and are comparable to other studies in the literature.

Our study has limitations. We included PET/CT scans performed within and outside our institution. This may have resulted in protocol variations. Despite this variation, a real-world phenomenon, our results equal or exceed those in the literature. Our sample size was small, and due to stringent inclusion criteria, we excluded several patients that further reduced our cohort size. Our study included patients treated with several chemotherapy combinations. However, almost all were platinum-based, and the majority combined with either paclitaxel or irinotecan. In this regard, it could be argued that the treatment regimens were relatively homogenous. The PET non-responders were grouped together as it was demonstrated in prior studies that the clinical outcomes in this cohort were similar, irrespective of the treatment regimen used [2]. In addition, not all patients in this series underwent surgery following chemoradiation; older patients who achieved a clinical complete response often deferred surgery. However, two phase III studies revealed no clear improvement in OS for surgery following chemoradiation, especially in patients with a clinical response [37,38]. There was class imbalance between various outcome groups which was addressed by using adaptive synthetic sampling. The decision to reduce the data to 64 gray levels for the CT data and 16 gray levels for the PET data is somewhat arbitrary. However, these are pragmatic choices. A reduced number of gray levels was chosen for the PET data due to the reduced spatial resolution which results in a lower pixel count for each tumor compared to the CT data. This ensures reasonable counting statistics whilst still maintaining a level of discriminatory power. Alternatively, the use of a fixed bin width may be appropriate here [38], but a satisfactory bin width, ensuring adequate counting statistics for all tumors, could not be determined for this dataset. These measures establish the robustness of our methods and perhaps will help in future studies when we seek external validation. A part of the segmentation was based on individual assessment; however, the inter-reader agreement was good to excellent. Finally, this was a retrospective study with its own inherent limitations, although most, if not all, radiomics or AI studies are retrospective.

## 5. Conclusions

This study demonstrates that PET/CT radiomics features in patients with locally advanced ESCC have the potential to predict clinical outcomes such as tumor and nodal status and PFS with greater accuracy than conventional anatomical or functional assessment. Radiomics models can also predict PET responders to induction chemotherapy. Before venturing into radiomics analysis of esophageal cancer, it is imperative that one considers histological variations with the inclusion of patients into the research, as well as avoids some of the basic pitfalls inherent to radiomics assessment. Future studies will be helpful for external validation of our model and evaluation of similar clinical outcomes in patients with adenocarcinoma.

## Figures and Tables

**Table 1:** Scanner and acquisition parameters.

**Scanners**	**Number of Scans**
GE Discovery 690	13
GE Discovery 710	5
GE Discovery LS	10
GE Discovery QX/i	2
GE Discovery ST	7
GE Discovery STE	14
Philips Gemini TF TOF 64	1
Siemens Biograph 40	3
Siemens Biograph 6	8
Siemens Emotion Duo	6
Siemens Sensation 16	5
**CT parameters**	**Median (range)**
kVp (kV)	130 (100–140)
Tube current (mA)	85 (35–305)
Matrix size	All at 512 × 512
In-plane resolution	0.977 (0.775–1.523)
Slice thickness	3.8 (3.0–5.0)
**PET parameters**	**Median (range)**
Matrix size	128 × 128 (128 × 128 to 484 × 484)
In-plane resolution (mm)	5.31 (1.03–5.47)
Slice thickness (mm)	3.3 (2.0–5.0)
Dose (MBq)	458 (320–788)
Uptake time (min)	65 (45–91)

**Table 2:** Patient characteristics.

Patient Characteristic	Male	Female	Total or *p*-Value
Total	51	23	74
Mean Age ± SD	65 (45–87)	66 (41–84)	0.852
Nodal Category (AJCC 8th)			
N0	6	7	0.049
N1/2	43	15	
Tumor Category (AJCC 8th)			
T2	7	1	0.201
T3/4	39	21	
PET Responders			
No	17	8	0.903
Yes	34	15	
Progression-Free Survival			
Yes	15	6	0.694
No	34	17	
Overall Survival (3 Yrs)			
Yes	20	10	0.831
No	29	13	
Induction Chemotherapy	51	23	74
Capecitabine/Oxaliplatin	0	1	
Carboplatin/Irinotecan	1	0	
Carboplatin/Paclitaxel	30	15	
Cisplatin/Irinotecan	18	5	
Cisplatin/Irinotecan/Docetaxel	1	1	
Docetaxel/Irinotecan	1	1	
Change in Chemo Regimen Post-Induction PET/CT			
Yes	10	6	16
No	41	17	58
SUV_max_	12.55 (10.01–15.64)	12.51 (9.32–16.64)	0.931

**Table 3:** Classifications of training and test datasets for different clinical parameters.

Clinical Parameters	Total	Training Cases	Test Cases
Nodal Category			
N0	13	7	6
N1/2	58	35	23
Tumor Category			
T2	8	5	3
T3/4	60	36	24
PET Responders			
Yes	49	29	20
No	25	15	10
Progression-Free Survival			
Yes	28	20	8
No	44	31	13
Overall Survival (3 Yrs)			
Yes	30	18	12
No	42	25	17

**Table 4:** Diagnostic accuracy of training and test datasets for various clinical parameters; 95% confidence intervals are presented within parentheses.

Clinical Parameter	Training CT Dataset	Test CT Dataset	Training PET Dataset	Test PET Dataset	Training Combined PET/CT Dataset	Test Combined PET/CT Dataset
Nodal Category	64.3 (51.9–75.4)	69.0 (49.2–84.7)	85.7 (75.3–92.9)	86.2 (68.3–96.1	87.1 (77.0–94.0)	86.2 (68.3–96.1)
Tumor Category	90.3 (81.0–96.0)	70.4 (49.8–86.3)	83.3 (72.7–92.1)	70.4 (49.8–86.3)	83.3 (72.7–91.1)	81.5 (61.9–93.7)
PET Responders	69.0 (55.5–80.5)	60.0 (40.6–77.3)	72.4 (59.1–83.3)	66.7 (47.2–82.7)	75.9 (62.8–86.1)	70.0 (50.6–85.3)
Progression-Free Survival	66.1 (53.0–77.7)	60.7 (40.6–78.5)	77.4 (65.0–87.1)	75.0 (55.1–89.3)	77.4 (65.0–87.1)	75.0 (55.1–89.3)
Overall Survival (3 Yrs)	56.0 (41.3–70.0)	51.7 (32.6–70.6)	58.0 (43.2–71.8)	55.2 (35.7–73.6)	68.0 (53.3–80.5)	62.1 (42.3–79.3)

## Data Availability

The datasets used and/or analyzed during the current study are available from the corresponding author on reasonable request.

## Supplementary Materials

The following supporting information can be downloaded at: www.mdpi.com/article/10.3390/cancers14123035/s1, Table S1: Jaccard index and dice similarity coefficient score for inter-reader agreement of the segmentations of the CT and PET datasets by two readers in twenty patients; Table S2: Intra-class correlation coefficients (ICC) calculated for all CT and PET datasets of radiomics features using a two-way mixed-effects model with single measures; Table S3: All diagnostic metrics predicting T category derived from radiomics models of CT, PET, and combined PET/CT training and test datasets; Table S4: All diagnostic metrics predicting N category derived from radiomics models of CT, PET, and combined PET/CT training and test datasets; Table S5: All diagnostic metrics predicting PET responders (defined as 35% reduction in SUVmax values on post-induction PET/CT) derived from radiomics models of CT, PET, and combined PET/CT training and test datasets; Table S6: All diagnostic metrics predicting progression-free survival derived from radiomics models of CT, PET, and combined PET/CT training and test datasets; Table S7: All diagnostic metrics predicting 3-year overall survival derived from radiomics models of CT, PET, and combined PET/CT training and test datasets. Table S8: Selected radiomics features for CT, PET and combined PET/CT for all outcomes.
