# Supplementary material for: Can 18F-FDG PET/CT Radiomics Features Predict Clinical Outcomes in Patients with Locally Advanced Esophageal Squamous Cell Carcinoma?
Source: Cancers (Basel). 2022 Jun 20;14(12):3035. doi: 10.3390/cancers14123035 (PMC9221147; doi:10.3390/cancers14123035)
Supplement: Supplementary file 1 [file cancers-14-03035-s001.zip › cancers-1778324-supplementary.pdf]

Supplemental Materials

Table S1. Jaccard Index and dice similarity coefficient score for inter-reader agreement of the segmentations of the CT and PET datasets by two readers in twenty patients.

Case No	Jaccard Index	Dice Score
**CT Datasets**		
1	0.337	0.504
2	0.679	0.809
3	0.701	0.824
4	0.560	0.718
5	0.711	0.831
6	0.564	0.721
7	0.725	0.841
8	0.534	0.697
9	0.657	0.793
10	0.815	0.898
11	0.608	0.756
12	0.714	0.833
13	0.677	0.807
14	0.465	0.635
15	0.531	0.694
16	0.658	0.794
17	0.756	0.861
18	0.684	0.812
19	0.680	0.810
20	0.673	0.804
**PET Datasets**		
1	0.679	0.809
2	0.887	0.940
3	0.680	0.809
4	0.697	0.822
5	0.832	0.909
6	0.710	0.831
7	0.834	0.909
8	0.669	0.801
9	0.786	0.880
10	0.828	0.906
11	0.795	0.886
12	0.878	0.935
13	0.708	0.829
14	0.381	0.551
15	0.851	0.920
16	0.758	0.862
17	0.872	0.932
18	0.851	0.920
19	0.583	0.737
20	0.691	0.817

Table S2. Intraclass correlation coefficients (ICC) calculated for all CT and PET datasets radiomic features using a two-way mixed effects model with single measures. Only parameters with good (0.60–0.79) or excellent (0.80–1.00) agreement were considered for subsequent predictive model development. As a result of ICC analysis, 11 CT radiomics features and 3 PET radiomics features were excluded from further analysis (excluded features in bold).

Radiomics Feature	Intraclass Correlation	
	CT	PET
min_FO	0.639	0.950
max_FO	**0.398**	1.000
mean_FO	0.813	0.986
range_FO	0.720	0.997
std_FO	0.749	0.995
var_FO	0.747	0.984
median_FO	0.969	0.972
skewness_FO	**0.346**	0.924
kurtosis_FO	**0.338**	0.935
entropy_FO	0.963	0.995
rms_FO	0.768	0.991
energy_FO	0.726	0.999
totalEnergy_FO	**0.482**	0.999
meanAbsDev_FO	0.852	0.993
medianAbsDev_FO	0.891	0.993
P10_FO	0.925	0.955
P90_FO	0.962	0.997
robustMeanAbsDev_FO	0.976	0.989
robustMedianAbsDev_FO	0.978	0.988
interQuartileRange_FO	0.982	0.987
coeffDispersion_FO	0.725	0.955
coeffVariation_FO	0.820	0.940
energy_GLCM	0.989	0.917
jointEntropy_GLCM	0.977	0.977
jointMax_GLCM	0.990	0.829
jointAvg_GLCM	0.636	0.953
jointVar_GLCM	0.743	0.946
contrast_GLCM	0.867	0.925
invDiffMom_GLCM	0.992	0.801
invDiffMomNorm_GLCM	0.701	0.940
invDiff_GLCM	0.992	0.865
invDiffNorm_GLCM	0.709	0.947
invVar_GLCM	0.958	0.726
dissimilarity_GLCM	0.950	0.945
diffEntropy_GLCM	0.960	0.925
diffVar_GLCM	0.845	0.934
diffAvg_GLCM	0.950	0.945
sumAvg_GLCM	0.636	0.953
sumVar_GLCM	0.728	0.947
sumEntropy_GLCM	0.959	0.959
corr_GLCM	0.684	0.942
clustTendency_GLCM	0.728	0.947
clustShade_GLCM	**0.592**	0.780
clustPromin_GLCM	**0.585**	0.937
haralickCorr_GLCM	0.839	0.931
autoCorr_GLCM	0.656	0.975
firstInfCorr_GLCM	0.895	0.976
secondInfCorr_GLCM	0.854	0.990
gln_RLM	0.993	0.972
glnNorm_RLM	0.972	0.829
glv_RLM	0.701	0.960

hglre_RLM	0.658	0.972
lglre_RLM	0.737	0.849
lre_RLM	0.997	0.716
lrhgle_RLM	**0.291**	0.966
lrlgle_RLM	0.941	0.801
re_RLM	0.850	0.950
rln_RLM	0.991	0.990
rlnNorm_RLM	0.994	0.712
rlv_RLM	0.998	0.746
rp_RLM	0.997	0.723
sre_RLM	0.993	0.688
srhgle_RLM	0.714	0.970
srlgle_RLM	0.654	0.854
sze_SZM	0.883	0.667
lze_SZM	0.981	0.706
gln_SZM	0.993	0.981
glnNorm_SZM	0.858	0.878
zln_SZM	0.983	0.993
zlnNorm_SZM	0.876	0.609
zp_SZM	0.988	0.706
lglze_SZM	0.730	0.836
hglze_SZM	0.672	0.964
szlgle_SZM	0.622	0.810
szhgle_SZM	0.726	0.905
lzlgle_SZM	0.965	0.635
lzhgle_SZM	**0.038**	0.949
glv_SZM	0.603	0.959
zlv_SZM	0.981	**0.596**
ze_SZM	0.827	0.983
lde_NGLDM	0.986	0.677
hde_NGLDM	0.998	0.716
lgce_NGLDM	0.745	0.849
hgce_NGLDM	0.656	0.972
ldlge_NGLDM	**0.534**	0.841
ldhge_NGLDM	0.818	0.887
hdlge_NGLDM	0.906	0.612
hdhge_NGLDM	**0.386**	0.922
gln_NGLDM	0.993	0.968
glnNorm_NGLDM	0.983	0.807
dcn_NGLDM	0.992	0.993
dcnNorm_NGLDM	0.996	**0.574**
glv_NGLDM	0.746	0.959
dcv_NGLDM	0.994	0.630
entropy_NGLDM	0.944	0.986
energy_NGLDM	0.969	0.965
coarseness_NGTDM	0.899	0.953
contrast_NGTDM	0.865	0.703
busyness_NGTDM	0.889	0.924
complexity_NGTDM	0.828	0.902
strength_NGTDM	**0.518**	0.988

Table S3. All diagnostic metrics predicting T category derived from radiomics models of CT, PET, and combined PET/CT training and test datasets. 95% confidence intervals are presented within parentheses.

Dataset	AUC	Sensitivity	Specificity	PPV	NPV	Accuracy
CT Training	0.89 (0.80 – 0.98)	80.6 (64.0 – 91.8)	100.0 (90.3 – 100.0)	100.0 (90.3 – 100.0)	83.7 (72.6 – 90.9)	90.3 (81.0 – 96.0)

CT Test	0.96 (0.87 – 1.00)	66.7 (44.7 – 84.4)	100.0 (74.6 – 100.0)	100.0 (74.6 – 100.0)	27.3 (17.6 – 39.8)	70.4 (49.8 – 86.3)
PET Training	0.90 (0.83 – 0.98)	80.6 (64.0 – 91.8)	86.1 (70.5 – 95.3)	85.3 (71.7 – 93.0)	81.6 (69.2 – 89.7)	83.3 (72.7 – 92.1)
PET Test	0.54 (0.15 – 0.93)	75.0 (53.3 – 90.2)	33.3 (0.8 – 90.6)	90.0 (79.7 – 95.4)	14.3 (2.8 – 44.8)	70.4 (49.8 – 86.3)
CT and PET Training	0.87 (0.77 – 0.97)	66.7 (49.0 – 81.4)	100.0 (90.3 – 100.0)	100.0 (90.3 – 100.0)	75.0 (65.4 – 82.6)	83.3 (72.7 – 91.1)
CT and PET Test	0.90 (0.79 – 1.00)	79.2 (57.9 – 92.9)	100.0 (74.6 – 100.0)	100.0 (74.6 – 100.0)	37.5 (21.6 – 56.7)	81.5 (61.9 – 93.7)

Table S4. All diagnostic metrics predicting N category derived from radiomics models of CT, PET, and combined PET/CT training and test datasets. 95% confidence intervals are presented within parentheses.

Dataset	AUC	Sensitivity	Specificity	PPV	NPV	Accuracy
CT Training	0.75 (0.63 – 0.86)	54.3 (36.7 – 71.2)	74.3 (56.7 – 87.5)	67.9 (52.7 – 80.0)	61.9 (51.9 – 71.0)	64.3 (51.9 – 75.4)
CT Test	0.65 (0.44 – 0.86)	73.9 (51.6 – 89.8)	50.0 (11.8 – 88.2)	85.0 (71.1 – 92.9)	33.3 (14.8 – 59.0)	69.0 (49.2 – 84.7)
PET Training	0.98 (0.95 – 1.00)	97.1 (85.1 – 99.9)	74.3 (56.7 – 87.5)	79.1 (68.2 – 86.9)	96.3 (78.9 – 99.5)	85.7 (75.3 – 92.9)
PET Test	0.90 (0.78 – 1.00)	100.0 (85.2 – 100.0)	33.3 (4.3 – 77.7)	85.2 (76.6 – 91.0)	100.0 (85.2 – 100.0)	86.2 (68.3 – 96.1)
CT and PET Training	0.93 (0.86 – 0.99)	82.9 (66.4 – 93.4)	91.4 (76.9 – 98.2)	90.6 (76.4 – 96.7)	84.2 (71.9 – 91.8)	87.1 (77.0 – 94.0)
CT and PET Test	0.90 (0.78 – 1.00)	91.3 (72.0 – 98.9)	66.7 (22.3 – 95.7)	91.3 (77.1 – 97.0)	66.7 (32.2 – 89.4)	86.2 (68.3 – 96.1)

Table S5. All diagnostic metrics predicting PET responders (defined as 35% reduction in SUVmax values on post induction PET/CT) derived from radiomics models of CT, PET, and combined PET/CT training and test datasets. 95% confidence intervals are presented within parentheses.

Dataset	AUC	Sensitivity	Specificity	PPV	NPV	Accuracy
CT Training	0.68 (0.54 – 0.83)	69.0 (49.2 – 84.7)	69.0 (49.2 – 84.7)	69.0 (55.1 – 80.1)	69.0 (55.1 – 80.1)	69.0 (55.5 – 80.5)
CT Test	0.51 (0.28 – 0.73)	60.0 (36.1 – 80.9)	60.0 (26.2 – 87.8)	75.0 (56.5 – 87.4)	42.9 (26.4 – 61.1)	60.0 (40.6 – 77.3)
PET Training	0.77 (0.65 – 0.90)	69.0 (49.2 – 84.7)	75.9 (56.5 – 89.7)	74.1 (58.9 – 85.1)	71.0 (57.8 – 81.4)	72.4 (59.1 – 83.3)
PET Test	0.65 (0.43 – 0.87)	70.0 (45.7 – 88.1)	60.0 (26.2 – 87.8)	77.8 (60.9 – 88.7)	50.0 (30.2 – 69.8)	66.7 (47.2 – 82.7)
CT and PET Training	0.84 (0.74 – 0.94)	72.4 (52.8 – 87.3)	79.3 (60.3 – 92.0)	77.8 (62.4 – 88.1)	74.2 (60.8 – 84.2)	75.9 (62.8 – 86.1)
CT and PET Test	0.57 (0.35 – 0.79)	75.0 (50.9 – 91.3)	60.0 (26.2 – 87.8)	79.0 (62.8 – 89.3)	54.6 (32.5 – 74.9)	70.0 (50.6 – 85.3)

Table S6. All diagnostic metrics predicting progression free survival derived from radiomics models of CT, PET, and combined PET/CT training and test datasets. 95% confidence intervals are presented within parentheses.

Dataset	AUC	Sensitivity	Specificity	PPV	NPV	Accuracy
CT Training	0.65 (0.51 – 0.79)	51.6 (33.1 – 69.9)	80.6 (62.5 – 92.6)	72.7 (54.6 – 85.5)	62.5 (52.7 – 71.4)	66.1 (53.0 – 77.7)
CT Test	0.71 (0.49 – 0.92)	62.5 (24.5 – 91.5)	60.0 (36.1 – 80.9)	38.5 (22.6 – 57.2)	80.0 (60.4 – 91.3)	60.7 (40.6 – 78.5)
PET Training	0.81 (0.70 – 0.92)	80.6 (62.5 – 92.6)	74.2 (55.4 – 88.1)	75.8 (62.7 – 85.3)	79.3 (64.5 – 89.0)	77.4 (65.0 – 87.1)
PET Test	0.66 (0.43 – 0.90)	50.0 (15.7 – 84.3)	85.0 (62.1 – 96.8)	57.1 (27.6 – 82.4)	81.0 (67.5 – 89.7)	75.0 (55.1 – 89.3)
CT and PET Training	0.81 (0.70 – 0.92)	64.5 (45.4 – 80.8)	90.3 (74.3 – 98.0)	87.0 (68.8 – 95.3)	71.8 (61.0 – 80.6)	77.4 (65.0 – 87.1)
CT and PET Test	0.73 (0.49 – 0.97)	50.0 (15.7 – 84.3)	85.0 (62.1 – 96.8)	57.1 (27.6 – 82.4)	81.0 (67.5 – 89.7)	75.0 (55.1 – 89.3)

Table S7. All diagnostic metrics predicting 3-year overall survival derived from radiomics models of CT, PET, and combined PET/CT training and test datasets. 95% confidence intervals are presented within parentheses.

Dataset	AUC	Sensitivity	Specificity	PPV	NPV	Accuracy
CT Training	0.55 (0.38 – 0.71)	44.0 (24.4 – 65.1)	68.0 (46.5 – 85.1)	57.9 (40.0 – 73.9)	54.8 (43.9 – 65.3)	56.0 (41.3 – 70.0)
CT Test	0.56 (0.34 – 0.79)	25.0 (5.5 – 57.2)	70.6 (44.0 – 89.7)	37.5 (15.0 – 67.2)	57.1 (46.0 – 67.6)	51.7 (32.6 – 70.6)
PET Training	0.62 (0.47 – 0.78)	56.0 (34.9 – 75.6)	60.0 (38.7 – 78.9)	58.3 (43.6 – 71.7)	57.7 (44.1 – 70.2)	58.0 (43.2 – 71.8)
PET Test	0.58 (0.36 – 0.80)	50.0 (21.1 – 78.9)	58.8 (32.9 – 81.6)	46.2 (27.8 – 65.7)	62.5 (45.5 – 76.9)	55.2 (35.7 – 73.6)
CT and PET Training	0.80 (0.68 – 0.92)	64.0 (42.5 – 82.0)	72.0 (50.6 – 87.9)	69.6 (53.3 – 82.1)	66.7 (52.9 – 78.1)	68.0 (53.3 – 80.5)
CT and PET Test	0.59 (0.37 – 0.81)	50.0 (21.1 – 78.9)	70.6 (44.0 – 89.7)	54.5 (32.2 – 75.2)	66.7 (51.2 – 79.2)	62.1 (42.3 – 79.3)

Table S8. Selected radiomics features for CT, PET and combined PET/CT for all outcomes

Dataset	1st Feature	2nd Feature	3rd Feature	4th Feature	5th Feature
T Category					
CT	size zone nonuniformity (SZM)	busyness (NGTDM)	small zone emphasis (SZM)	interquartile range (FO)	coefficient of dispersion (FO)
PET	cluster shade (GLCM)	small zone low gray level emphasis (SZM)	low dependency low gray level emphasis (NGLDM)	small zone high gray level emphasis (SZM)	gray level non-uniformity (NGLDM)
CT/PET	size zone nonuniformity (CT SZM)	busyness (CT NGTDM)	small zone emphasis (CT SZM)	cluster shade (PET GLCM)	coefficient of dispersion (CT FO)
N Category					
CT	interquartile range (FO)	coarseness (NGTDM)	robust median absolute deviation (FO)	robust mean absolute deviation (FO)	coefficient of dispersion (FO)
PET	minimum (FO),	10th percentile (FO)	complexity (NGTDM)	correlation (GLCM)	sum variance (GLCM)
CT/PET	minimum (PET FO)	complexity (PET NGTDM)	10th percentile (PET FO)	correlation (PET GLCM)	coefficient of variation (CT FO)
PET Responder					
CT	coefficient of dispersion (FO)	small zone emphasis (SZM)	size zone nonuniformity (SZM)	2nd information measure of correlation (GLCM)	long run low gray level emphasis (RLM)
PET	sum variance (GLCM)	cluster tendency (GLCM)	low dependence high gray level emphasis (NGLDM)	Haralick correlation (GLCM)	gray level variance (SZM)
CT/PET	coarseness (CT NGTDM)	minimum (PET FO)	size zone non-uniformity normalized (CT SZM)	run emphasis (PET RLM)	cluster shade (PET GLCM)
PFS					
CT	busyness (NGTDM)	size zone non-uniformity (SZM)	large zone low gray level emphasis (SZM)	energy (FO)	coefficient of variation (FO)
PET	difference variance (GLCM)	energy (NGLDM)	coarseness (NGTDM)	entropy (NGLDM)	difference entropy (GLCM)
CT/PET	difference variance (PET GLCM)	energy (PET NGLDM)	inverse difference moment normalized (CT GLCM)	contrast (PET NGTDM)	low gray level run emphasis (CT RLM)
OS					
CT	coefficient of variation (FO)	second information measure of correlation (GLCM)	correlation (GLCM)	busyness (NGTDM)	low gray level count emphasis (NGLDM)
PET	complexity (NGTDM)	gray level variance (RLM)	gray level variance (NGLDM)	joint variance (GLCM)	cluster tendency (GLCM)
CT/PET	gray level variance (PET SZM)	cluster prominence (PET GLCM)	2nd information measure of correlation (CT GLCM)	difference entropy (PET GLCM)	correlation (CT GLCM)
